# 

**Published:** 1905-01

**Authors:** 


### BOOKS RECEIVED.

Diseases of the Liver, Gallbladder, and Bile Ducts. By H. D. Rolleston, A.M., M.D., (Cantab.), F.R.C.P., Physician to Saint George's Hospital, London; formerly Examiner in Medicine at the University of Durham, England. Octavo volume of 794 pages, fully illustrated, including seven colored insert plates. Philadelphia, New York, London: W. B. Saunders & Company. 1904. (Cloth, $6.00 net.)

Normal Histology and Microscopical Anatomy. By Jeremiah S. Ferguson, M.Sc., M.D., Instructor in Normal Histology, Cornell University Medical College, New York. Octavo, pp. 758. With 462 illustrations, many in colors. New York and London: D. Appleton & Company. 1905.

A Laboratory Manual of Human Anatomy. By Lewellys F. Barker, M.B., Tor. Professor of Anatomy in the University of Chicago and Rush Medical College. Octavo, pp. 583. Illustrated. Philadelphia and London: J. B. Lippincott Company. 1904. (Price, $5.00.)

A Manual of Personal Hygiene. Proper Living upon a Physiologic Basis. By American Authors. Edited by Walter L. Pyle, A.M., M.D., Assistant Surgeon to the Wills Eye Hospital, Philadelphia. Second edition, revised and enlarged. Duodecimo volume of 441 pages, fully illustrated. Philadelphia, New York, London: W. B. Saunders & Company. 1904. (Bound in silk, $1.50 net.)

Saunders's Medical Hand-Atlases. Atlas and Epitome of General Pathologic Histology. By Dr. H. Dürck, of Munich. Edited, with additions, by Ludvig Hektoen, M.D., Professor of Pathology, Rush Medical College, Chicago. With 172 colored figures on 77 lithographic plates, 36 text-cuts, many in colors, and 371 pages of text. Philadelphia, New York, London: W. B. Saunders & Company. 1904. (Cloth, $5.00 net.)

Diet in Health and Disease. By Julius Friedenwald, M.D., Clinical Professor of Diseases of the Stomach in the College of Physicians and Surgeons, Baltimore; and John Ruhräh, M.D., Clinical Professor of Diseases of Children in the College of Physicians and Surgeons, Baltimore. Octavo volume of 689 pages. Philadelphia, New York, London: W. B. Saunders & Company. 1904. (Cloth, $4.00 net.)

Gallstones and Their Surgical Treatment. By B. G. A. Moynihan, M.S. (Lond.), F. R. C. S., Senior Assistant Surgeon to Leeds General Infirmary, England. Octavo volume of 386 pages, illustrated with text-cuts, some in colors, and nine colored insert plates. Philadelphia, New York, London: W. B. Saunders & Company. 1904. (Cloth, $4.00 net.)

Progressive Medicine, Volume VI., December, 1904. A Quarterly Digest of Advances, Discoveries and Improvements in the Medical and Surgical Sciences. Edited by Hobart Amory Hare, M.D., Professor of Therapeutics and Materia Medica in the Jefferson Medical College of Philadelphia. Octavo, 374 pages, illustrated. Philadelphia and New York: Lea Brothers & Co., Publishers. (Per annum, in four cloth-bound volumes, $9.00; in paper binding, $6.00, carriage paid to any address.)

Transactions of the American Otological Society. Thirty-seventh Annual Meeting held at Atlantic City, N. J., July 11 and 12, 1904. F. L. Jack, M.D., Secretary. Published by the Society.

